# ADP Ribosylation Factor Like 2 (Arl2) Regulates Breast Tumor Aggressivity in Immunodeficient Mice

**DOI:** 10.1371/journal.pone.0007478

**Published:** 2009-10-15

**Authors:** Anne Beghin, Stéphane Belin, Rouba Hage Sleiman, Stéphanie Brunet Manquat, Sophie Goddard, Eric Tabone, Lars P. Jordheim, Isabelle Treilleux, Marie-France Poupon, Jean-Jacques Diaz, Charles Dumontet

**Affiliations:** 1 Inserm, U590, Lyon, France; 2 Université Lyon 1, ISPB, Lyon, France; 3 CNRS, Centre de Génétique Moléculaire et Cellulaire, UMR 5534, Villeurbanne, France; 4 Centre Léon Bérard, Service Anatomie-Cytologie Pathologiques, Lyon, France; 5 Institut Curie, Paris, France; Health Canada, Canada

## Abstract

We have previously reported that ADP ribosylation factor like 2 (Arl2), a small GTPase, content influences microtubule dynamics and cell cycle distribution in breast tumor cells, as well as the degree and distribution of phosphorylated P53. Here we show, in two different human breast adenocarcinoma models, that Arl2 content has a major impact on breast tumor cell aggressivity both *in vitro* and *in vivo*. Cells with reduced content of Arl2 displayed reduced contact inhibition, increased clonogenic or cluster formation as well as a proliferative advantage over control cells in an *in vitro* competition assay. These cells also caused larger tumors in SCID mice, a phenotype which was mimicked by the *in vivo* administration of siRNA directed against Arl2. Cells with increased Arl2 content displayed reduced aggressivity, both *in vitro* and *in vivo*, with enhanced necrosis and were also found to contain increased PP2A phosphatase activity. A rt-PCR analysis of fresh human tumor breast samples suggested that low Arl2 expression was associated with larger tumor size and greater risk of lymph node involvement at diagnosis. These data underline the role of Arl2, a small GTPase, as an important regulator of breast tumor cell aggressivity, both *in vitro* and *in vivo*.

## Introduction

Arl2 is a GTPase belonging to the ADP ribosylation factor (ARF) family [1], [2]. Several genetic studies suggest that Arl2 plays a role in MT dynamics [3]–[5]. In mammalian cells, Bhamidipati *et al.* have demonstrated direct binding between Arl2 and tubulin binding cofactor D (TBC-D), a protein involved in tubulin folding [6]. TBC-D interacts with beta tubulin and contributes to the production of polymerizable tubulin heterodimers. TBC-D can also induce the dissociation of soluble alpha/beta heterodimers [7]–[10]. Binding of Arl2 to TBC-D inhibits the heterodimer-dissociating activity of TBC-D [6]. More recently, it has been reported that Arl2 inhibited TBC-D-dependent cell dissociation from the monolayer and disassembly of the apical complex [11].

The entire cytosolic pool of Arl2 is complexed with TBC-D and with the heterotrimeric protein phosphatase 2A (PP2A) [12]. PP2A is one of the major serine/threonine phosphatases of mammalian cells. It is composed of three subunits: a regulatory A subunit (PP2Aa), various types of regulatory B subunits (PP2Ab) and a catalytic C subunit (PP2Ac). By its involvement in different signal transduction pathways, PP2A plays a crucial role in the regulation of several fundamental cell processes such as cell cycle progression [13], DNA replication [14], apoptosis [15] and protein synthesis. PP2A is considered to behave as a tumor suppressor protein [14]. Mutations affecting PP2A genes have been found in lung, breast and colon carcinoma [16], [17], as well as in cervical, ovarian, gastric and nasopharyngeal carcinomas. These mutations frequently consist in deletions in the 11q23 region that encodes the A subunit [16], [17]. Furthermore, inhibitors of PP2A such as okadaic acid or microcystin-LR are known to promote cell proliferation and tumorigenesis [12], [18], [19].

Using breast adenocarcinoma derived MCF7 and MDA-MB 231 cells expressing different levels of Arl2, we have shown that alterations of cellular Arl2 protein content were associated with modifications of polymerization-competent −alpha/beta tubulin heterodimer levels resulting in altered MT dynamic properties and with modifications of mitotic progression as well as with modifications of the content, localization and activity of PP2Ac with no significant changes in PP2Ac mRNA levels [20]. We also showed that Arl2 content was associated with the degree and distribution of phosphorylated P53, in particular phosphor-ser15-P53, a form which was preferentially bound to microtubules [21].

In this study, we show that Arl2 content is a major determinant of aggressive phenotype and tumorigenicity in two different breast cancer cell models, and low Arl2 expression levels appear to be correlated with enhanced aggressivity in the clinic.

## Results

### Contact inhibition of cancer cells is dependent on Arl2 expression levels

**Figure 1:**
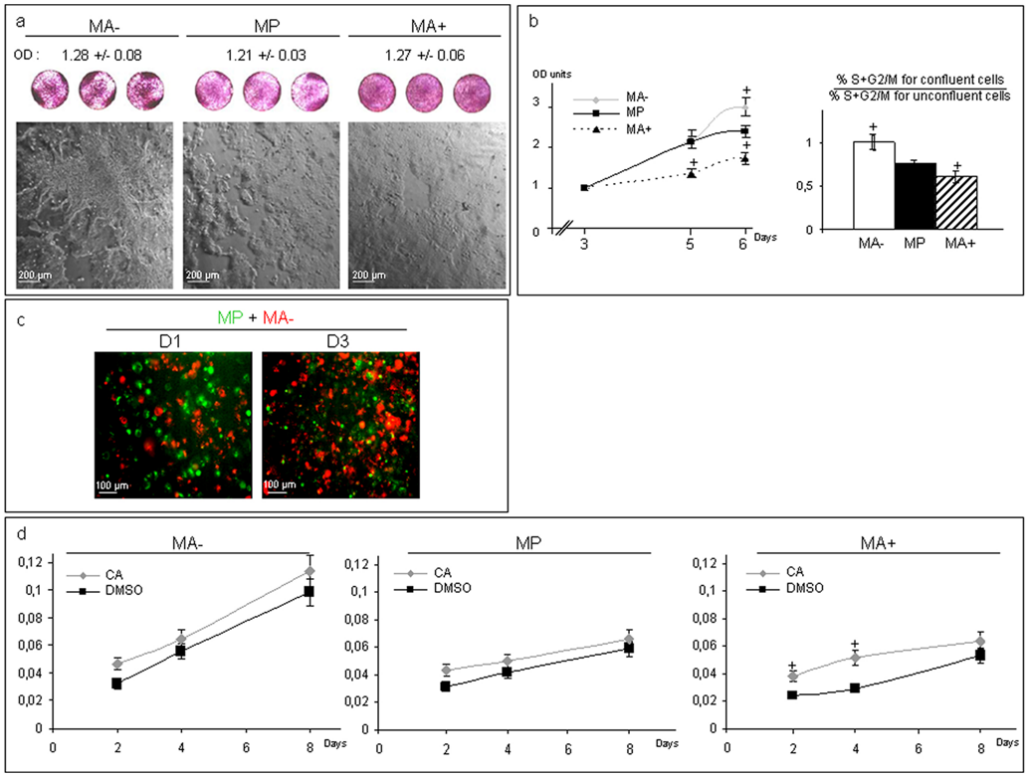
Arl2 content alterations induce modification of proliferation and contact inhibition behavior of cancer cells. A: Upper: MA-, MP and MA+ cells were visualized after MTT incubation and before dissolvation of formazan crystals (purple dishes) to observe cell surface occupation. Formazan crystals were then dissolved and optic density (OD) measured to obtain the control of cells seeding (in arbitrary units). Lower: Phase contrast microscopy observations of MA-, MP and MA+ cells. B: Left: Means of proliferation rates of MA−, MP and MA+ cells evaluated by MTT assay expressed in optic density units per day. Right: Means of percentages of confluent cells versus non-confluent cells in S+G2-M phases evaluated by flow cytometry. Bars represent standard deviations. Statistical significance was determined using Student's t test (p<0.05). C: Coculture cell competition assay using different fluorescent vital cell linker dyes, PKH 67 (green) for MP cells and PKH 26 (red) for MA-. Distributions of each cellular population were followed using a fluorescent microscope from day 1 to day 3 after cell seeding (representative images). D: Means of proliferation rates of MA−, MP and MA+ cells incubated with vehicle (DMSO) or with cantharidic acid (CA), evaluated by MTT assay expressed in optic density units (OD units) per day. Bars represented standard deviations. Statistical significance was determined using Student's t test.

To study the contact inhibition of the cell lines expressing different levels of Arl2 we have evaluated their respective proliferation rates at confluence using phase contrast microscopy, MTT assays and flow cytometry analyses. For these experiments the cellular seeding concentrations were adjusted according to morphological differences of these cell lines previously described [20]. The biggest cells (MA+ and MdaA+) were seeded at a 30% lower concentration whereas the smallest cells (MA- and MdaA-) were seeded at a 30% higher concentration than the respective control cells (MP and MdaP). Phase contrast microscopy observations were used to analyse the cell distribution in Petri dishes (Figure 1a). MA- cells presented a heterogeneous surface occupation with some unoccupied spaces along with high cellular density regions (Figure 1a). Conversely MP, and most notably MA+ cells, occupied available space more evenly with a homogeneous distribution. In addition MA- cells were able to form multi-layers while MP and MA+ grew only in mono-layers, suggesting that MA- cells had lost contact inhibition (Figure 1a).

The proliferation rates were evaluated using MTT assays at 3, 5 and 6 days post seeding. Three days of culture corresponded to the confluence conditions for the three cell lines (Figure 1b). The proliferation rates of both MA- and MP were higher than that of MA+ whatever the time of culture (p<0.05). The proliferation rate of MA- and MP cells were very similar until 5 days of culture whereas that of MA- cells was significantly increased (p<0.05) compared to that of MP cells after 5 days of culture. Finally flow cytometry analysis (Figure 1b) showed that the ratio of cells in S-G2/M phase at confluence versus cells in exponential growth was significantly lower (−14%, p<0.05) in MA+ cells and higher (+22%, p<0.05) in MA- cells in comparison to that of control MP cells. Similar results were obtained for MDA-MB 231 derived models (data not shown). These data confirmed that cells with reduced Arl2 content continued to grow at confluence whereas cells with increased Arl2 content were particularly sensitive to contact inhibition.

Co-culture experiments with MP and MA- cells were performed using specific fluorescent linker dyes to follow each cell population (Figure 1c). After 3 days of co-culture, MA- cells (red fluorescent dye) have proliferated more quickly than MP cells (green fluorescent dye) under confluent conditions, confirming the strong proliferative behavior of MA- cells, and its relative growth advantage over control cells. Similar results were obtained using a green fluorescent dye for MA- cells and a red dye for MP cells (dye swap experiment, data not shown).

Altogether these results showed that, at confluence, cells expressing high levels of Arl2 (MA+ and MdaA+) displayed a stronger growth arrest with increased contact inhibition whereas cells with reduced Arl2 content (MA- and MdaA-) did not seem to undergo growth arrest and maintained growth ability even at confluence.

Since Arl2 interacts with PP2A and the content of Arl2 influences PP2A activity [20], we sought to determine whether the proliferation rates of the different cell lines could be related to PP2A activity. Growth rates of each cell line were measured in the presence of cantharidic acid, a well characterized inhibitor of PP2A. In the presence of cantharidic acid at a dose selectively inhibiting PP2A (IC50 for PP2A activity  = 40 nM and for PP1  = 473 nM, manufacturer's indications) (Figure 1d), the growth rate of MA+ cells was significantly enhanced (+30–70%, p<0,05) whereas those of MA- and MP cells were not significantly modified. This result suggests that the proliferation rate is related to PP2A activity, since cells with the highest Arl2 and PP2A contents were the most prone to proliferate in the presence of a PP2A inhibitor.

### Effect of Arl2 content on three-dimensional cell growth

**Figure 2:**
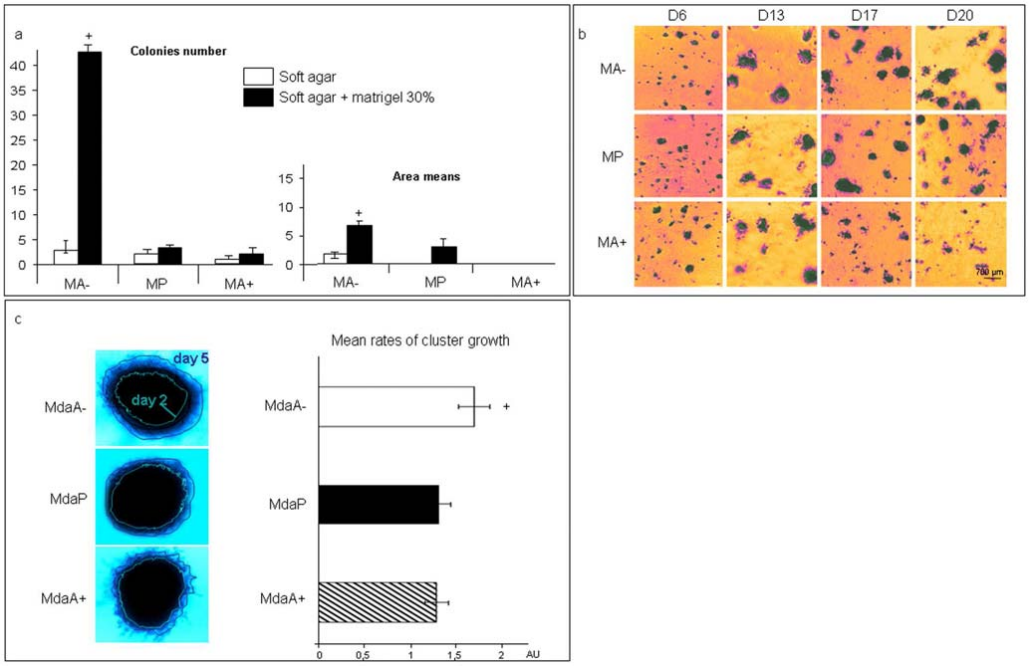
Effect of Arl2 content on three-dimensional cell growth. A: Soft agar and soft agar complemented with matrigel (30% v/v) assay for MA-, MP and MA+ cells expressed in colonies number (left) and in area means (right). Bars represented standard deviations. Statistical significance was determined using Student's t test. B: Follow up of MA-, MP and MA+ cells overlayed on matrigel. Representative images displayed cellular colonies growth from day 6 to Day 20 after cells seeding. C: Matrigel cluster assay performed for MdaA-, MdaP and MdaA+ cells. Light blue lines represent the contour of cells clusters at day 2 and dark blue lines at day 5 after cells seeding (left, representative images). Mean rates of cluster growth were evaluated by daily measurements of cell clusters areas from day 2 to day 5. Bars represented standard deviations. Statistical significance was determined using Student's t test.

Experiments using soft agar and soft agar complemented with 30% Matrigel (extracellular matrix) were performed in order to distinguish between anchorage-free growth ability (soft agar) and clonogenic behavior (soft agar+Matrigel) (Figure 2a). No significant differences were observed between MA-, MP and MA+ cells grown in soft agar alone. However, in the soft agar complemented with 30% of Matrigel, MA+ cells developed very few (less than 3 per well) and smaller colonies after 26 days of growth in comparison with MP cells, whereas MA- cells developed significantly more (>40 per well, p<0.05) and bigger (+50% p<0.05) colonies than MP cells (Figure 2a). There results suggested that MA- cells have a stronger, and MA+ cells a weaker, clonogenic potential than MP cells.

MA-, MP and MA+ were also seeded on a Matrigel coat to study their proliferative behavior in a three-dimensional extracellular matrix (Figure 2b). After 6 to 13 days of growth, the three cell lines displayed similarly growth properties with formation of small colonies. After 17 days of growth, the MA+ colonies began to disaggregate. After 20 days of growth, MP colonies also began to disaggregate whereas MA- colonies were still present, exhibiting a compact mass (Figure 2b).

These assays were not applicable to the MDA-MB 231 derived cell lines because these cells did not develop isolated cellular colonies in Matrigel or soft agar. We therefore studied the three-dimensional growth capacity of MDA-MB 231 derived cells with the Matrigel cluster assay (Figure 2c). This assay consists in loading a compact cell cluster in a thick layer of Matrigel in order to follow the growth capacity of a “tumor-like” cluster in a three dimensional environment. For the first two days, no significant differences between the three cell lines were observed. After day 2, MdaA- cell clusters had a significant higher (+30%, p<0.05) growth rate than MdaP and MdaA+ cell clusters.

### Arl2 content influences tumor growth *in vivo*

We evaluated the tumorigenicity of the different MDA-MB 231 and MCF7-derived cell lines in SCID mice. MdaA-, MdaP, MdaA+ cells were transfected with a plasmid encoding firefly luciferase as described in the materials and methods section. Resulting bioluminescent cell lines (MdaA-.luc, MdaP.luc, MdaA+.luc) with bioluminescence *in vitro* were injected into the mammary fat pad of SCID mice and monitored for tumor growth.

**Figure 3:**
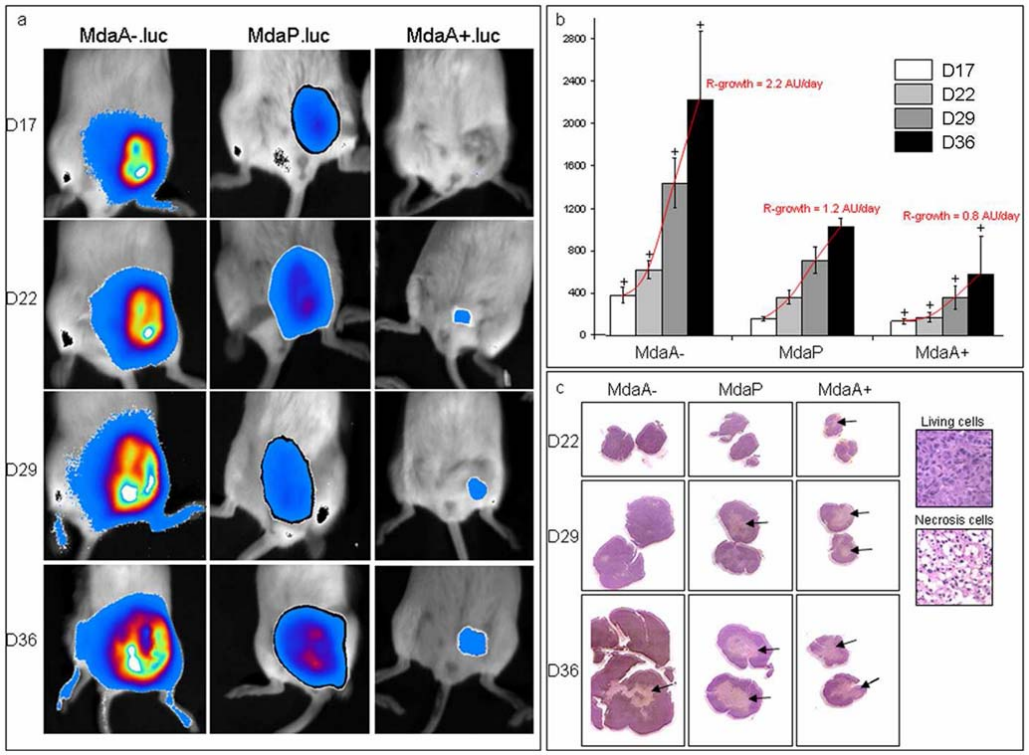
Arl2 content influences tumorigenesis. A: Representative images of non invasive bioluminescent follow up (from day 17 to day 36) for a same tumor of each cell lines (MdaA-.luc, MdaP.luc, MdaA+.luc) developed from mice mammary fat pad injected cells. B: Mean rates of tumoral bioluminescence in mice expressed in photons/sec for each day and sublines. Tumor growths (R-growths) were evaluated using a linear projection of the growth curve (red) and expressed in arbitrary units per day (AU/day). Bars represent standard deviations. Statistical significance was determined using Student's t test. C: Representative images of histochemistry of MdaA-, MdaP, MdaA+ cells tumors slides at day 22, 29 and 36. Black arrows show necrotic regions (in light pink) which are particularly important in MdaA+ cells tumors.

Bioluminescent measurements showed that the MdaA-.luc derived tumors were significantly larger (+65% to 80%, p<0.05) than those developed by MdaP.luc cells (Figure 3a). Conversely, MdaA+.luc derived tumors were significantly smaller (−45% to 55%, p<0.05) than those formed by MdaP.luc cells, resulting in a large difference between MdaA-.luc and MdaA+.luc derived tumors (+100–200%, p<0.01). In addition, the mean rate of tumor growth (R-growth) was evaluated between days 17 and 36 (Figure 3b). Tumors derived from MdaA-.luc cells presented a two-fold higher R-growth than MdaP.luc tumors which displayed a 35% higher R-growth than MdaA+.luc tumors. Similar results were obtained in MCF7 and non-bioluminescent MDA-MB 231 derived cells (data not shown).

Tumors were excised from the mice after euthanasia (Figure 3c). Histopathology analyses showed that all MdaA+.luc tumors presented a large necrotic area as early as day 22 post-injection. These areas encompass 15% of the total tumor volume on day 22 and more than 70% on day 36. Conversely, MdaA-.luc tumors, although much larger, contained much less necrotic areas, representing a maximum of 25% of total tumor volume on day 36. MdaP.luc tumors displayed intermediate size necrosis. Similar results were obtained with MA-, MP and MA+ cells (data not shown). These differences in spontaneous necrosis were likely to account at least in part for the differences observed in tumor growth.

To determine whether PP2A activity was significantly different according to Arl2 status during tumor growth *in vivo*, we measured PP2A activity in tumors. As shown in Table 1, MA+ and MdaA+ cells displayed stronger phosphatase activity against the phosphoserine substrate than their corresponding variants, MP and MdaP. In MA- and MdaA- cells, PP2A activity was similar to that of the control cells. These results suggest that Arl2 status modulates PP2A activity *in vivo*.

**Table 1:** PP2A activity measured using a phosphoserine substrate in Arl2 variant lines.

	PP2A activity
MA-	1,021±266
MP	984±204
MA+	1,352±160
MdaA-	1,303±82
MdaP	1,466±35
MdaA+	2,006±67

Values represent the picomoles of phosphate issued after the dephosphorylation of the above substrates and are denoted as mean ± standard deviation.

### Inhibition of Arl2 *in vivo* enhances tumor growth and development of human breast cancer xenografts

**Figure 4:**
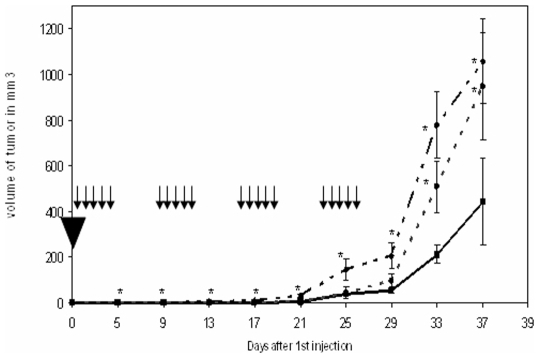
Effect of *in vivo* siRNA directed against Arl2 or PP2A on human MDA-MB-231 xenografts. SCID mice were injected subcutaneously on day 1 (black triangle) with MDA-MB 231 cells then treated daily, 5 days a week for 4 weeks with siRNA (scrambled continous line), directed against Arl2 (dotted line), directed against PP2A (dashed line). *Significant differences were observed between the groups receiving scrambled and Arl2 or PP2A directed siRNA (p<0.05). Arrows represent siRNA injections.

We assessed the effect of Arl2 and PP2A inhibition *in vivo* on the tumor growth of MDA-MB 231 cells. For this, we administered duplex siRNAs directed against each of these targets intraperitoneally five days a week for four weeks. Scrambled siRNA was used as a control. Both siRNA against Arl2 and against PP2A were found to enhance tumor growth in comparison to controls injected with scrambled siRNA (Figure 4). These results strongly suggest that Arl2 profoundly influences tumor cell aggressivity *in vivo*. As previously shown, inhibition of PP2A was also associated with enhanced tumor aggressivity.

To confirm *in vivo* modulation of expression of target genes, tumor and liver samples were obtained while mice were receiving siRNA therapy. As shown in Table 2, gene expression of PP2A and Arl2 was significantly and specifically repressed by the corresponding siRNA both in liver and in tumors during therapy.

**Table 2:** Expression levels of Arl2 and PP2A of tumor and liver samples in mice exposed to control siRNA, anti PP2A siRNA or anti Arl2 siRNA.

	PP2A (rt-PCRunits)	PP2A (rt-PCRunits)	Arl2 (rt-PCR units)	Arl2 (rt-PCR units)
	Tumor	Liver	Tumor	Liver
Control siRNA	0.37±0.22	0.47±0.16	5.98±0.66	0.80±0.11
Arl2 siRNA	0.38±0.06	0.30±0.02	0.23±0.11*	0.03±0.02*
PP2A siRNA	0.06±0.03*	0.06±0.01*	3.94±4.15	0.61±0.11

* : significantly different from control.

### Expression levels of Arl2 in human primary breast tumors

Thirty eight primary breast tumors were available for analysis. Arl2 mRNA content was determined after normalization with 18S ribosomal RNA. Five primary tumors were associated with lymph node involvement and four tumors were of larger size (T3 or T4 of the TNM classification). When classifying tumors according to Arl2 expression in primary tumors (lower or greater than median in the entire group) we found that all of the large tumors (p = 0.05, Chi-square test) and all of the tumors with lymph node metastases (p = 0.03, Chi-square test) were found in samples with low Arl2 expression in the primary tumor (Table 3). Although these observations should be confirmed on larger series, these data suggest that tumors with low Arl2 content are more aggressive clinically.

**Table 3:** Tumor size and lymph node involvement in primary human breast tumors according to Arl2 expression levels.

	Tumor size	Tumor size	Node involvement	Node involvement
	T1/T2	T3/T4	Negative	Positive
Low Arl2	12	4	11	5
High Arl2	13	0	13	0

Arl2 levels were determined by quantitative rt-PCR on frozen primary breast tumors.

## Discussion

Our results suggest that Arl2 content influences breast tumor growth and aggressivity through a PP2A mediated pathway. In a previous study we have demonstrated that Arl2 content influences the content and the activity of the catalytic subunit of PP2A (PP2Ac) [20]. Given the reported tumor suppressor properties of PP2A [14], we have determined the impact of Arl2 on tumor cell aggressivity and tumorigenic capacity in two breast cancer cell models, MCF7 and MDA-MB-231. We observed that modifications of Arl2 expression levels could induce modifications of contact inhibition, clonogenic potential, and tumor growth, including spontaneous apoptosis of tumor cells. Preliminary data also suggest that cells with reduced Arl2 content possess enhanced metastatic potential when injected orthotopically in SCID mice (data not shown). We have thus shown for the first time, in two different breast cancer cell models, that a reduced Arl2 expression level is associated with a more aggressive neoplastic phenotype. These results were supported by the observation that low Arl2 content was associated with greater aggressivity in primary human breast tumors.

Cells with reduced Arl2 content were found to behave more aggressively, with loss of contact inhibition, ability to grow in multilayer *in vitro*, enhanced clonogenic or cluster formation potential, gain of survival advantage vis-à-vis their normal counterparts in an *in vitro* competition assay as well as enhanced tumor growth and reduced spontaneous necrosis tumors *in vivo*. The role of Arl2 downregulation in the acquisition of an aggressive phenotype is supported by similar results observed when mice injected with parental cells were treated with siRNA against Arl2. This treatment effectively modulated Arl2 gene expression in tumors and was associated with increased tumor growth. Conversely increased expression yielded the opposite phenotype, both *in vitro* and *in vivo*, with reduced clonogenic potential and reduced tumor growth with massive spontaneous tumor necrosis. These results therefore strongly suggest that Arl2 content is correlated with *in vitro* and *in vivo* aggressivity in the two breast cancer models studied.

PP2A has been shown to play an important inhibitory effect on cell proliferation [22], [23]. We have previously shown that the content and the activity of the catalytic subunit of PP2A (PP2Ac) is modified in cells with altered Arl2 content [20]. Here we confirm that inhibition of PP2A in SCID mice is associated with increased tumor aggressivity. Analysis of enzymatic activity in Arl2 modified tumors showed that PP2A activity was increased in cells with increased Arl2 content, a result in keeping with our observation that these cells proliferated more actively in the presence of a PP2A inhibitor. PP2A is known to interact directly with many proteins and modify their activity by either activating or inhibiting them. The anti-apoptotic proteins Bcl2 and Bcl-xl and the pro-apoptotic proteins such as Bax are targets of PP2A. In these proteins, the dephosphorylation occurs at the serine residues and shifts the signaling pathway toward apoptosis by activating and deactivating the pro- and anti-apoptotic proteins, respectively [24]. The increased PP2A activity of cells with high Arl2 content is thus likely to explain the massive apoptosis observed in tumors derived from these cells. Conversely the enhanced aggressivity observed in cells with reduced Arl2 content could not be attributed to a reduction in PP2A enzyme activity.

It remains to be established which are the key regulators involved in Arl2/PP2A regulation of tumor aggressivity. PP2A dephosphorylates a large variety of substrates involved in cell cycle regulation. Our previous study found that alterations in Arl2 content profoundly modified microtubule dynamics as well as the duration of the various phases of mitosis, in particular anaphase and telophase [20]. We have also reported that Arl2 content influences the content and distribution of phosphor-ser15-P53 [21]. Moreover, other authors have recently demonstrated the involvement of MT dynamics and of PP2A in the regulation of concentration of E-cadherin at cell-cell contacts. Stehbens *et al.* identified a pool of MTs that extend radially into cell-cell contacts, blocking MT dynamics and thereby altering the ability of cells to concentrate and accumulate E-cadherin at cell-cell contacts [25]. Takahashi *et al.* have shown that treatment of the cells with inhibitors of PP2A caused disruption of cell-cell adhesion and proposed that PP2A could play a crucial role in the maintenance of cell-cell adhesion [26].

In conclusion these data suggest that Arl2, a small GTP protein whose role is yet largely unknown, appears to be a significant regulator of PP2A content and activity in breast cancer cells, both *in vitro* and *in vivo*. In preclinical models, reduced Arl2 content is associated with enhanced tumor aggressivity while increased Arl2 content is associated with reduced aggressivity and enhanced spontaneous necrosis. In primary human breast tumors, low Arl2 mRNA content is associated with larger tumor size and greater risk of lymph node involvement at diagnosis. These data suggest that Arl2, possibly through a PP2A-mediated pathway, is a key regulator of breast tumor aggressivity.

## Materials and Methods

### Ethics Statement

All research involving animals have been conducted according to relevant national and international guidelines. The protocol was approved by the Lyon Animal Experimental Committee and animals were treated in accordance with European Union guidelines for laboratory animal care and use.

### Cell culture and transfection

All cell lines were grown in DMEM containing L-glutamine, penicillin (200 IU/ml), streptomycin (200 µg/ml), and fetal bovine serum (10%) at 37°C in the presence of 5% CO2. MCF-7 cells were stably transfected with empty pcDNA3 (MP cells) or pcDNA3 containing sense Arl2 (MA+ cells) or antisense Arl2 (MA- cells) as describe elsewhere [20]. The same experimental strategy was used to obtain stable transfectants from MDA-MB 231 cells (commercial cell lines ATCC number HTB26), yielding MdaP, MdaA+, and MdaA- cells. MdaP, MdaA+ and MdaA-, cells were then co-transfected with plasmids expressing the firefly luciferase gene (pGL3-basic, Promega) and the puromycin resistance gene (pSuper/Puro; Oligoengine) using lipofectamine (Invitrogen) according to the manufacturer's recommendations, and bioluminescent puromycin resistant cells were then selected. The three bioluminescent derivatives cells named, MdaP.luc, MdaA+.luc and MdaA-.luc were used for *in vivo* studies.

### Determination of confluent cell proliferation rates

Cell proliferation was estimated using the methylthiazoletetrazolium (MTT) assay. Twenty thousand cells were seeded per well of a 24-well plate and incubated at 37°C. Every 24 h up to 6 days, MTT (500 µg/per well) was added to 3 wells of each plate. After 2 h of incubation at 37°C, supernatants were removed and absorbance measured as described previously [27].

### Cell cycle distribution by flow cytometry analysis

Cells were collected and incubated at 4°C during 2 h with 1 ml of propidium iodide solution (0.05 mg/ml) containing Nonidet-P40 (0.05%). Cells were analyzed using a FACS Calibur flow cytometer (BD Biosciences Europe, Erembodegem, Belgium) and Modfit LT 2.0™ software (VeritySoftware Inc, Topsham, United States).

### *In vitro* cell growth assays

All microscopic analyses were performed in the Centre Commun de Quantimétrie (Université de Lyon I, France), unless otherwise stated.

#### Coculture cell competition assay

MP and MA- cells were separately incubated with different fluorescent cell linker dyes (respectively PKH 67 green and PKH 26 red (Sigma)) following the manufacturer's recommendations and were then placed in coculture at a 1∶1 ratio. A similar dye-swap experiment was performed with PKH 67 green for MA- cells and PKH 26 red for MP cells. Fluorescent images were taken at day 1 and 3 after cell.

#### Growth in soft-agar and soft-agar containing matrigel

At the time of plating in soft-agar in 6-well plates, 2×10^4^ total cells were mixed with 1.5 ml of 0.45% low melting point (LMP) agarose-DMEM (top layer) and then poured on top of 1.5 ml of solidified 0.75% LMP agarose-DMEM (bottom layer) completed with 30% (v/v) matrigel (BD Biosciences) for the matrigel-containing soft-agar condition. Colonies were counted and photographed after 26 days in triplicate.

#### Overlay three-dimensional culture on matrigel

Overlay three-dimensional cultures on matrigel (BD Biosciences) were performed as described previously in triplicate [28]. 100 µl of matrigel were added in each well of an 8-well glass chamber slide. For each cell line, a cellular suspension containing 12,500 cells/ml in a medium containing 4% (v/v) matrigel was prepared. 400 µl of this mixture was plated per well on top of the solidified matrigel. This corresponds to a final overlay solution of 5000 cells/well in medium containing 2% matrigel. Cell colony formation was monitored using microscopic observation and image acquisition up to 20 days after incubation.

#### Matrigel cluster assay

For each of the MDA-MB 231 derived cell lines, a cellular suspension containing 1×10^6^ cells/µl in DMEM medium was prepared. Matrigel (BDBiosciences) was added to the wells of an 8-well glass chamber slide in a volume of 300 µl. Before matrigel polymerization, 1 µl containing 1×10^6^ cells was carefully loaded in the middle of the matrigel coating. Then, matrigel containing preparations were allowed to solidify at 37°C and cells loading were formed a compact crowd of cells. 150 µl of complete medium were then added on wells containing matrigel. Area of each cells cluster was determined for up to 5 days using microscopic observation and quantified using ImageJ software. Measurements were performed in triplicate.

### Orthotopic tumorigenicity assays

For each cell line, five female SCID mice aged 4–6 weeks were anesthetized and injected with 50 µl of 2×10^6^ cells suspended in 50% DPBS/50% matrigel into the abdominal mammary fat pad. The size of the tumor was measured twice a week by external measurement or *in vivo* bioluminescent imaging. Mice were killed when the tumors reached a greatest diameter of 1,2 cm. The protocol was approved by the Lyon Animal Experimental Committee and animals were treated in accordance with European Union guidelines for laboratory animal care and use.

*In vivo* bioluminescent imaging was performed by administering the substrate D-luciferin by intraperitoneal injection at 150 mg/kg in D-PBS (Invitrogen), and anesthetized (1–3% isoflurane). Mice were then placed inside a light-tight box under a photon counting camera (NightOWL II LB 983, Berthold Technologies, Germany) with continuous exposure to 1–2% isoflurane + 4% oxygene. Regions of interest from displayed images were identified around the tumor sites and were quantified as photons/s using WinLight^32^ software (Berthold Technologies, Germany) with background bioluminescence substraction. Tumor growth (R-growth) was evaluated using a linear projection of the growth curve.

### Pathological analysis of tumor clusters

After fixation in Bouin's solution the tumor or cell cluster was dehydrated with alcohol, immersed in xylene and embedded in paraffin. Four µm-thick slides were dried at 58°C for 30 minutes in an incubator. After deparaffination and rehydration, the paraffin sections were stained with hematoxylin and eosin. The slides were mounted with a xylene mounting medium.

### siRNA administration *in vivo*

The siRNA were purchased from Sigma-Aldrich as duplex desalted and deprotected non-modified sequences of 21 base pairs. The sequences were the following: Arl2, 5′-AACCCUCCUCAUCUUUGCUAA-TT; PP2Ac, 5′- GAGGUUCGAUGUCCAGUUATT-TT, scrambled (SCR), 5′- GCUGAUAGCAUGGUCUGAUTT-TT.

In this experiment, three groups of ten SCID mice were studied. The groups I, II and III correspond respectively to the scrambled siRNA, Arl2 siRNA and PP2Ac siRNA the injected mice. The first day, 3 millions of MDA-MB 231 cells were implanted subcutaneously in all mice followed by intraperitoneal injections of SCR, PP2A and Arl2 siRNAs (4 µ in 50 µl of PBS). The injections of siRNAs were continued daily for a period of four weeks and the volumes of tumors were monitored twice per week till two weeks after the end of injections.

### Quantitative real-time RT-PCR

The level of inhibition exhibited by the Arl2 and PP2A siRNA on their respective targets was assessed by real-time quantitative PCR on tumors and livers extracted 24 hours after the last siRNA injection. Total mRNA extraction, reverse transcription and real-time quantitative RT-PCR were performed using a lightcylcer thermal cycler (Roche, Manheim, Germany). Forward and reverse primer sequences used for Arl2 were respectively 5′- GGCTCCTGACCATTCTGAAG and 3′- TGTAGTTCTGGGACCTCGTG; for PP2Ac: 5′- CCCTGGATCGTTTACAGGAA and 3′- ATGGTAAAAGTCACGTGGGT. Results were analyzed with RelQuant software (Roche, Manheim, Germany).

### PP2A enzyme activity assay

The specific activity of the phosphatase PP2A was assessed using a system based on the immunoprecipitation of PP2Ac (Upstate-Millipore, USA) as recommended by the manufacturer by using 300 µg of total protein of each of the cell lines MA-, MP, MA+, MdaA-, MdaP, MdaA+. The peptide containing a phosphorylated threonine residue (K-R-pT-I-R-R) provided with the system was replaced by a similar peptide containing a phophoserine residue (K-R-pS-I-R-R), synthesized by Dr. Ficheux (IBCP Université Lyon 1) in order to better mimick the physiological situation. The optical density of the products issued from the dephosphorylation was read as an absorbance at 620 nm. The samples were compared to a standard curve corresponding to a range of 0 to 2000 pmol of free phosphates. The values obtained for the cells MP and Mda P were used as reference.

### PCR analysis of primary breast tumors

Primary breast tumors were obtained from the Centre Léon Bérard anti Cancer Tissue Bank. Patients gave written informed consent for these analyses. Total mRNA was extracted with Trizol reagent (Invitrogen, Cergy Pontoise, France) and two micrograms were converted into cDNA by Moloney leukaemia virus reverse transcriptase (Invitrogen) as described in the manufacturer's manual. cDNA levels were normalized to the expression of 18S ribosomal gene using the pre-developed TaqMan assay reagents control kit (Applied biosystem, Foster City, Canada) and assayed for Arl2 by real time PCR performed in a LightCycler thermal cycler (Roche, Meylan, France) as previously described [20].
