# Correction: ADP Ribosylation Factor Like 2 (Arl2) Regulates Breast Tumor Aggressivity in Immunodeficient Mice

**DOI:** 10.1371/annotation/b0d43779-c9aa-44fe-a46d-71d7d2bc4a6a

**Published:** 2009-11-16

**Authors:** Anne Beghin, Stéphane Belin, Rouba Hage-Sleiman, Stéphanie Brunet Manquat, Sophie Goddard, Eric Tabone, Lars P. Jordheim, Isabelle Treilleux, Marie-France Poupon, Jean-Jacques Diaz, Charles Dumontet

The third author's name was spelled incorrectly. The correct name is: Rouba Hage-Sleiman. The correct citation is: Beghin A, Belin S, Hage-Sleiman R, Brunet Manquat S, Goddard S, et al. (2009) ADP Ribosylation Factor Like 2 (Arl2) Regulates Breast Tumor Aggressivity in Immunodeficient Mice. PLoS ONE 4(10): e7478. doi:10.1371/journal.pone.0007478 

